# A multi-centre, double-blind, placebo-controlled, randomised trial of combination methotrexate and gefitinib versus methotrexate alone to treat tubal ectopic pregnancies (GEM3): trial protocol

**DOI:** 10.1186/s13063-018-3008-6

**Published:** 2018-11-20

**Authors:** James May, Colin Duncan, Ben Mol, Siladitya Bhattacharya, Jane Daniels, Lee Middleton, Catherine Hewitt, Arri Coomarasamy, Davor Jurkovic, Tom Bourne, Cecilia Bottomley, Alexandra Peace-Gadsby, Ann Doust, Stephen Tong, Andrew W. Horne

**Affiliations:** 1 Simpsons Centre for Reproductive Health, Royal Infirmary of Edinburgh, Edinburgh, UK; 2 MRC Centre for Reproductive Health, Queen’s Medical Research Institute, University of Edinburgh, 47 Little France Crescent, Edinburgh, EH16 4SA UK; 3 Monash Health, Monash Medical Centre, Melbourne, Australia; 4 Obstetrics and Gynaecology, Division of Applied Clinical Sciences, University of Aberdeen, Aberdeen Maternity Hospital, Aberdeen, UK; 5 Nottingham Clinical Trials Unit, Nottingham Health Science Partners, Queen’s Medical Centre, Nottingham, UK; 6 Birmingham Clinical Trials Unit, Institute of Applied Health Research, College of Medical and Dental Sciences, University of Birmingham, Birmingham, UK; 7 Tommy’s National Centre for Miscarriage Research, Birmingham Women’s Hospital, Birmingham, UK; 8 Gynaecology Diagnostic and Treatment Unit, University College Hospital, London, UK; 9 Obstetrics and Gynaecology, Chelsea and Westminster NHS Hospital Foundation Trust, London, UK; 10 Queen Charlotte’s and Chelsea Hospital, London, UK; 11 Ectopic Pregnancy Trust, London, UK; 12 University of Melbourne, Mercy Hospital for Women, Melbourne, Australia

**Keywords:** Gynaecology, Reproductive medicine, Ectopic pregnancy, Gefitinib, Epidermal growth factor receptor, Methotrexate, Surgery, Clinical trial

## Abstract

**Background:**

Tubal ectopic pregnancy (tEP) is the most common life-threatening condition in gynaecology. Treatment options include surgery and medical management. Stable women with tEPs with pre-treatment serum human chorionic gonadotrophin (hCG) levels < 1000 IU/L respond well to outpatient medical treatment with intramuscular methotrexate. However, tEPs with hCG > 1000 IU/L can take significant time to resolve with methotrexate and require multiple outpatient monitoring visits. In pre-clinical studies, we found that tEP implantation sites express high levels of epidermal growth factor receptor. In early-phase trials, we found that combination therapy with gefitinib, an orally active epidermal growth factor receptor antagonist, and methotrexate resolved tEPs without the need for surgery in over 70% of cases, did not cause significant toxicities, and was well tolerated. We describe the protocol of a randomised trial to assess the efficacy of combination gefitinib and methotrexate, versus methotrexate alone, in reducing the need for surgical intervention for tEPs.

**Methods and analysis:**

We propose to undertake a multi-centre, double-blind, placebo-controlled, randomised trial (around 70 sites across the UK) and recruit 328 women with tEPs (with pre-treatment serum hCG of 1000–5000 IU/L). Women will be randomised in a 1:1 ratio by a secure online system to receive a single dose of intramuscular methotrexate (50 mg/m^2^) and either oral gefitinib or matched placebo (250 mg) daily for 7 days. Participants and healthcare providers will remain blinded to treatment allocation throughout the trial. The primary outcome is the need for surgical intervention for tEP. Secondary outcomes are the need for further methotrexate treatment, time to resolution of the tEP (serum hCG ≤ 15 IU/L), number of hospital visits associated with treatment (until resolution or scheduled/emergency surgery), and the return of menses by 3 months after resolution. We will also assess adverse events and reactions until day of resolution or surgery, and participant-reported acceptability at 3 months.

**Discussion:**

A medical intervention that reduces the need for surgery and resolves tEP faster would be a favourable treatment alternative. If effective, we believe that gefitinib and methotrexate could become standard care for stable tEPs.

**Trial registration:**

ISRCTN Registry ISRCTN67795930. Registered 15 September 2016.

**Electronic supplementary material:**

The online version of this article (10.1186/s13063-018-3008-6) contains supplementary material, which is available to authorized users.

## Background

Tubal ectopic pregnancy (tEP) is a significant contributor to maternal morbidity and mortality in both the developed and developing world [1, 2]. Health economic models suggest that medical management with a single intramuscular injection of methotrexate (MTX) is a cost-effective treatment for haemodynamically stable women with tEP, but there is a significant risk of treatment failure if the pre-treatment human chorionic gonadotrophin (hCG) levels are > 1500 IU/L. Treatment failure carries a subsequent risk of emergency laparoscopic surgical excision (with its inherent risks of damage to visceral organs and impact on subsequent fertility) [3]. In addition, women with higher hCG levels take a significant time to resolve with MTX and require multiple outpatient monitoring visits. Women with lower pre-treatment serum hCG levels (< 1000 IU/L) can be managed with MTX, or by expectant management without medical intervention [4, 5]. There exists a need for more effective medical treatments for tEP with higher hCG levels to reduce the need for emergency surgery and to reduce the time to resolution associated with MTX management.

Gefitinib is an orally active epidermal growth factor receptor (EGFR) antagonist licensed to treat non-small cell lung cancer [6]. In pre-clinical studies, we found that tEP implantation sites express high levels of EGFR and that gefitinib augments MTX-induced regression of pregnancy-like tissue [7]. To translate this into clinical care, we performed a phase I, single-arm, open-label, dose-escalation trial (GEM1) administering a combination of 250 mg oral gefitinib (one dose (*n* = 3), three daily doses (*n* = 3), and seven daily doses (*n* = 6)) and intramuscular MTX (50 mg/m^2^) to 12 women with tEP [8]. Resolution (fall in serum hCG to ≤ 15 IU/l) with combination therapy (up to seven doses of oral gefitinib with the MTX) was faster than the median time for tEPs to resolve with MTX alone when compared with historic controls (21 vs 32 days). We then carried out a phase II, single-arm, open-label trial (GEM2) administering a combination of 7 days of 250 mg oral gefitinib and intramuscular MTX (50 mg/m^2^) to 28 women with tEP [9]. The trial has been successfully completed [10].

MTX is an established standard in the treatment of tEP, and adverse reactions such as stomatitis and nausea are usually mild and self-limiting. More severe adverse reactions are rare but include hepatotoxicity, myelosuppression, and nephrotoxicity. Regarding the safety of gefitinib, data from post-marketing surveillance representing over 92,000 patients suggest that EGFR inhibitors are largely free of serious side effects (Food and Drug Administration (FDA) report) [11]. Of note, the data on tolerability are based on patients taking gefitinib daily on an ongoing, indefinite basis, after primary treatment of lung cancer. Diarrhoea and skin rash are the most common side effects (20–30%). The skin rash, described as acneiform, can be severe, but is generally self-limited. These side effects are quite common; in GEM1 we noted that 67% of women developed a transient acneiform rash and 42% had diarrhoea [8].

### Objectives

The primary objective of this trial is to compare the efficacy of a combination of gefitinib and MTX to treat tEP (hCG ≥ 1000 IU/L and ≤ 5000 IU/L) with MTX alone in terms of the need for emergency (rescue) surgery. In our secondary objectives, we will evaluate the need for a second dose of MTX, time to resolution of pregnancy (serum hCG ≤ 15 IU/L), number of associated hospital visits, time of return to menses, and the safety, tolerability, and acceptability of the combination treatment.

## Methods

### Study design

This will be a phase III, multi-centre, double-blind, placebo-controlled, randomised trial in around 70 UK early pregnancy units. At the time of writing (19 May 2018), GEM3 is recruiting from 54 sites but expansion to 70 sites is in progress. The protocol described in the manuscript is an abbreviated version. The full protocol used by investigators can be found at https://www.birmingham.ac.uk/research/activity/mds/trials/bctu/trials/womens/gem3/index.aspx. This protocol has been prepared in accordance with Standard Protocol Items: Recommendations for Interventional Trials (SPIRIT), and a checklist is provided as Additional file 1.

### Inclusion and exclusion criteria

Women diagnosed with a haemodynamically stable tEP diagnosed on ultrasound with serum hCG concentrations ≥ 1000 IU/L and ≤ 5000 IU/L and deemed suitable for MTX treatment by their attending clinical team as per local protocols will be screened for eligibility for the GEM3 trial against the criteria listed in Table 1.

**Table 1:** Inclusion and exclusion criteria

Inclusion criteria	
Diagnosis of either; 1. Definite tubal ectopic pregnancy (EP) (extra-uterine gestational sac with yolk sac and/or embryo, without cardiac activity on ultrasound scan (USS)); or 2. Clinical decision of probable tubal EP (extrauterine sac-like structure or inhomogeneous adnexal mass on USS with a background of sub-optimal serum human chorionic gonadotrophin (hCG) concentrations (on at least 2 different days)	
Clinical decision made for treatment of tubal EP with methotrexate (MTX)	
Able to understand all information (written and oral) presented (using an interpreter if necessary) and provide signed consent	
18–50 years of age at time of randomisation	
Pre-treatment serum hCG level of 1000–5000 IU/L (within 1 calendar day of randomisation)	
Clinically stable	
Haemoglobin between 100 and 165 g/L no more than 3 calendar days before randomisation	
Able to comply with treatment and willing to participate in follow-up	
Exclusion criteria	
Pregnancy of unknown location (PUL)	
Evidence of intra-uterine pregnancy	
Breastfeeding	
Hypersensitivity to gefitinib	
EP mass on ultrasound greater than 3.5 cm (mean dimensions)	
Evidence of significant intra-abdominal bleed on USS defined by echogenic free fluid above the uterine fundus or surrounding ovary within 1 calendar day of treatment	
Significant abdominal pain, guarding/rigidity	
Clinically significant abnormal liver/renal/haematological indices noted no more than 3 calendar days before randomisation	
Galactose intolerance	
Significant dermatological disease, e.g. severe psoriasis/eczema	
Significant pulmonary disease, e.g. severe/uncontrolled asthma	
Significant gastrointestinal illness, e.g. Crohn’s disease/ulcerative colitis	
Participating in any other clinical trial of an investigational medicinal product	
Previous participation in GEM3	
Japanese ethnicity	

### Participant enrolment

Potential participants will be identified by a member of their clinical care team and provided with a patient information sheet. All eligible women will have the opportunity to discuss the trial with a member of the clinical research team. Consent will be taken once the patient has had ample time to read the patient information sheet and had her questions answered. Consent will be obtained by a member of staff who has had specific trial training and Good Clinical Practice (GCP) training, or targeted trial-specific GCP training.

### Participant log

**Fig. 1:**
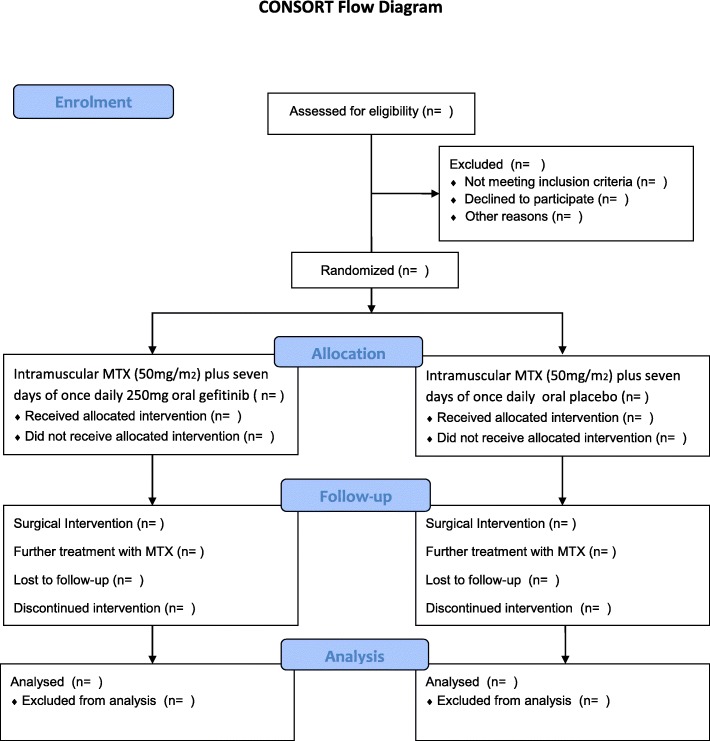
CONSORT flow diagram. MTX methotrexate

The clinical research team will keep an electronic log of women who fulfil the eligibility criteria including those who are invited to participate in the study, women recruited, and those who leave the trial early. Reasons for non-recruitment (e.g. non-eligibility, refusal to participate) will be recorded. We will attempt to collect reasons for non-participation from women who decline to take part. No further information will be collected on ineligible women (Fig. 1).

### Randomisation and blinding

Participants will be randomised individually into the GEM3 trial in an equal ratio of MTX/gefitinib or MTX/placebo. Randomisation will be performed via a secure web-based service provided by the Birmingham Clinical Trials Unit (BCTU). A minimisation procedure using a computer-based algorithm will be used to avoid chance imbalances in baseline serum hCG concentrations (< 1500 IU/L, ≥ 1500 to < 2500 IU/L, ≥ 2500 IU/L), body mass index (BMI; < 25, ≥ 25), tEP size (< 2 cm, ≥ 2 cm), and recruiting centre. To avoid any possibility of the treatment allocation becoming too predictable, a random factor will be included within the algorithm whereby allocation to the minimised treatment group will occur with a probability of < 1. The BCTU will electronically manage the drug supply via a bespoke database. Following randomisation and allocation, the relevant staff will be emailed with the trial identifier, the date of treatment, and the treatment bottle allocation.

### Intervention

Randomised women will be given a single-dose injection of intramuscular MTX (50 mg/m^2^) with seven daily oral doses of gefitinib or matched placebo (250 mg). No changes in dose will be permitted. The study treatment will be started on the same day as the MTX is administered with the remaining treatment being taken in the participant’s home. Participants will be asked to take the study drug at approximately the same time each day. The placebo and active drug will appear identical, thus blinding trial participants, care providers, and outcome assessors.

### Outcomes and assessments

**Fig. 2:**
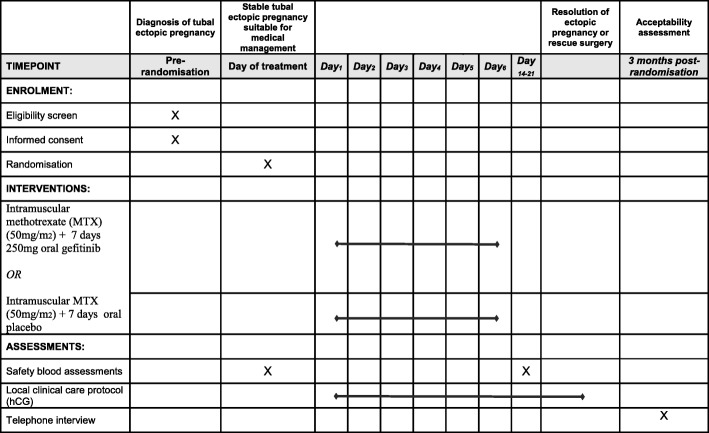
Schedule of enrolment, interventions, and assessments. hCG human chorionic gonadotrophin

A summary of study assessments and time points is given in Fig. 2. Our primary outcome is surgical intervention for treatment of the index EP (salpingectomy/salpingostomy by laparoscopy/laparotomy).

The secondary outcomes are:

1. The need for a second dose of MTX.
2. Number of days to resolution of tEP. Resolution is defined by serum hCG levels falling to non-pregnancy levels (hCG ≤ 15 IU/L), which corresponds to a negative urinary pregnancy test using the most sensitive assays.
3. Number of treatment-associated hospital visits until resolution or emergency ‘rescue’ surgery
4. Return to menses, assessed 3 months post-resolution by telephone interview.
5. Safety and tolerability: women will be assessed clinically (at each contact as per local policies) and biochemically (haematological, renal, and liver function tests between days 14–21 post-treatment) and these will be repeated if deemed clinically significant.
6. Acceptability of treatment: assessed 3 months post-resolution by participant-reported Likert scores via a telephone interview.

Participants will be asked at each visit if they have taken the drug so that the researcher can record how much study drug has been taken.

### Sample size

We have calculated the sample size based on data taken from our GEM2 study [12], our published cohort data [13, 14], and an audit of Edinburgh and Imperial of women undergoing usual care (unpublished data 2012; focusing on tEP patients with serum hCG > 1000 IU/L). The cohort and audit data suggest a 30% rate of surgical intervention in the MTX group, with 15% expected in the gefitinib plus MTX group (actual figure from GEM2 was 14% but this has been conservatively rounded up). To detect this absolute difference in proportions of 15% with 90% power and an alpha error rate of 5%, a total of 322 participants would need to be randomised (161 per group). In our retrospective Scottish cohort of 397 women, 2 were lost to follow-up (0.5%) [14]. In a national audit of 222 Scottish women, no women were lost to follow-up (unpublished data 2007; Ectopic Pregnancy: Scottish Outcomes with Methotrexate; Scottish Programme for Clinical Effectiveness in Reproductive Health). Assuming and adjusting for a 2% loss to follow-up rate, 328 participants need to be recruited.

### Statistical analysis

A separate statistical analysis plan, providing a detailed description of the planned analysis, has been prepared and reviewed by the Data Monitoring Committee (DMC), and a brief outline is described here. Analysis will be by intention to treat. Every attempt will be made to gather data on all women randomised, irrespective of compliance with the treatment protocol. Point estimates, 95% confidence intervals and *p* values from two-sided tests will be calculated for all main outcome measures. We will use a mixed effects log-binomial regression model adjusting for the minimisation variables (all fixed effects apart from centre which will be a random effect) to calculate the relative risk and 95% confidence for the primary outcome; a chi-squared test will be used to determine statistical significance of the group parameter in the model. In the situation where we have convergence issues with the log binomial we will revert to a Poisson regression model incorporating robust standard errors. Other dichotomous secondary outcomes (e.g. need for additional treatment) will be analysed in the same fashion as the primary outcome. Time from randomisation to successful resolution will be analysed using a Cox proportional hazards model. Standard methods will be used to analyse other outcomes. Tests for statistical heterogeneity will be performed prior to any examination of effect estimate within subgroups. Sensitivity analyses will be performed on the primary outcome to investigate the impact of missing data.

### Data management and monitoring

Information will be extracted from the clinical record into the case report form (CRF). Data will be collected as it becomes available, i.e. at or shortly after each patient visit by members of the clinical team supported by research staff. We will collect reasons for missing data. All data generated from the study will be stored anonymously in a bespoke database created by the BCTU. Participants will be allocated a unique number. All data will be stored on a Birmingham University server with limited access in accordance with the Data Protection Act (UK). During the period of recruitment to the study, interim analyses of major endpoints will be supplied, in strict confidence, to an independent DMC along with updates on results of other related studies, and any other analyses that the DMC may request. The DMC will advise the chair of the Trial Steering Committee (TSC) if, in their view, any of the randomised comparisons in the trial have provided both (a) “proof beyond reasonable doubt” that for all, or for some, types of patient one particular treatment is definitely indicated or definitely contraindicated in terms of a net difference in the major endpoints, and (b) evidence that might reasonably be expected to influence the patient management of many clinicians who are already aware of the other main trial results. Appropriate criteria of proof beyond reasonable doubt cannot be specified precisely, but a difference of at least *p* < 0.001 (similar to Haybittle-Peto stopping boundary) in an interim analysis of a major endpoint may be needed to justify halting, or modifying, the study prematurely. If this criterion were to be adopted, it would have the practical advantage that the exact number of interim analyses would be of little importance, so no fixed schedule is proposed. The TSC can then decide whether to close or modify any part of the trial. Unless this happens, however, the central trial management group, the TSC, the investigators, and all of the central administrative staff (except the statisticians who supply the confidential analyses) will remain unaware of the interim results.

### Safety assessment

Information on adverse events will be collected at each contact with the participants during routine clinical appointments. Any serious adverse events (SAEs) that occur will be reported to the trial sponsor (University of Edinburgh and NHS Lothian) and Astra Zeneca according to their respective timelines and followed up until resolution of the event. In-patient observation and surgical treatment for EP will not be considered a SAE, nor will any planned hospitalisation or hospitalisation for pre-existing conditions. These will be recorded as adverse events (AEs) but will not be reported as SAEs. All AEs and SAEs will be recorded from the time a participant signs the consent form to take part in the study until resolution. Participants will be asked to return any unused drug to the research team for safe disposal in the pharmacy. All participants will be given an emergency card to carry while participating in the study. The mechanism for code breaking will include an online code break facility which will be part of the BCTU randomisation database with restricted access as to who can break the blind. The sponsor will have access to the randomisation database in case of potential serious unexpected serious adverse reactions (SUSARs).

### Ethics and dissemination

This trial was externally peer reviewed by the UK National Institute for Health Research (NIHR) as part of the process of obtaining funding from the Efficacy and Mechanistic Evaluation (EME) programme. Ethical approval was obtained from the Scotland A Research Ethics Committee (16/SS/0014) on 29 February 2016. Data will be presented at international conferences and published in peer-reviewed journals. We will make the information available to the public through national bodies and charities, e.g. the Ectopic Pregnancy Trust.

## Discussion

If effective, we believe that this combination gefitinib and MTX could become the standard of care for stable tEPs. We believe that many clinicians would agree that an intervention that is shown to be significantly better than MTX in reducing the risk of surgery (our primary outcome) and resolves tEPs faster would be a better treatment alternative. The avoidance of surgery in the management of tEPs avoids surgical and anaesthetic risk for the patient, and is likely to result in cost saving for healthcare services. There is limited evidence regarding the impact on fertility of treatment options for tEPs; however, previous randomised controlled trials have not identified significant differences in fertility rates following medical or surgical treatment [15, 16].

In this randomised trial, we have chosen endpoints that are highly clinically relevant and eligibility criteria that reflect clinical practice in patients undergoing medical treatment of tEP and, as such, we believe that the results will be generalisable. Allocation will be strictly random and trial participants, care providers, and outcome assessors will be blinded to the intervention. We anticipate high ascertainment in terms of the primary outcome. We acknowledge the trial’s limitations, including the potential for unblinding that may happen if participants experience side effects associated with gefitinib, notably the skin rash. The decision for intervention will be made by individual sites and there may exist variation in the propensity to intervene between sites.

The post-marketing surveillance data regarding gefitinib supports its safety in terms of a side-effect profile as well as tolerability. Of particular note, interstitial lung disease (ILD) is a very rare but a serious side effect of gefitinib. It is a thickening of the lung parenchyma that can be fatal in a third of cases. Of the 31,045 patients in the USA who took gefitinib (reported to the FDA), 84 developed ILD (0.3%). However, the median length of time that gefitinib was taken for those who developed ILD was 42 days. In contrast, we plan to administer 250 mg gefitinib tablets orally, one daily for only 7 days, in addition to MTX. This is an extremely short duration of treatment compared with gefitinib’s current marketing indications and existing data usage. Furthermore, it is unclear whether ILD also occurred in these patients taking gefitinib indefinitely because of the presence of concurrent lung cancer. There have been almost no reports of ILD occurring among patients taking Cetuximab (a neutralising monoclonal epidermal growth factor antibody) to treat colon cancer. Thus, we believe the probability of our cohort developing this condition is negligible.

In our first trial (GEM1), we presented encouraging data to suggest this treatment option held promise. We compared six participants with a pre-treated serum hCG 1000–3000 IU/L and treated with gefitinib and MTX with 71 historical controls who presented to our clinical service (with the same serum hCG range) and were treated with MTX alone [8]. The serum hCG levels among those treated with the combination fell considerably more precipitously than those treated with MTX alone and the tEPs resolved 34% faster. We also successfully treated eight participants with extra-tubal EPs with combination gefitinib and MTX. Finally, we have completed a single arm trial of 28 women with tEPs who had a pre-treatment serum hCG between 1000 and 10,000 IU/L [10]. However, all these were single-arm, open-label trials. As such, we have embarked on this large randomised trial to demonstrate that adding gefitinib to MTX is significantly more effective than MTX alone. It has been designed with a primary outcome that is highly clinically relevant.

### Trial status

Currently recruiting. Trial start date 2 November 2016. Anticipated recruitment end date is December 2019.

Protocol version 6, 12 April 2017.

See https://www.birmingham.ac.uk/research/activity/mds/trials/bctu/trials/womens/gem3/index.asp.

## Additional file

Additional file 1: SPIRIT 2013 checklist: recommended items to address in a clinical trial protocol and related documents. (DOC 125 kb)

## Abbreviations

EGFR: Epidermal growth factor receptor
hCG: Human chorionic gonadotrophin
MTX: Methotrexate
tEP: Tubal ectopic pregnancy
